# Efficacy and safety of glecaprevir/pibrentasvir in patients with chronic HCV infection and psychiatric disorders: An integrated analysis

**DOI:** 10.1111/jvh.13110

**Published:** 2019-05-20

**Authors:** David Back, Pamela Belperio, Mark Bondin, Francesco Negro, Andrew H. Talal, Caroline Park, ZhenZhen Zhang, Brett Pinsky, Eric Crown, Federico J. Mensa, Fiona Marra

**Affiliations:** ^1^ University of Liverpool Liverpool UK; ^2^ U.S. Department of Veterans Affairs VA Palo Alto Healthcare System Palo Alto California; ^3^ AbbVie Inc North Chicago Illinois; ^4^ University of Geneva Geneva Switzerland; ^5^ Jacobs School of Medicine and Biomedical Sciences University of Buffalo Buffalo New York

**Keywords:** chronic hepatitis C, drug interactions, mental disorders, sustained virologic response, treatment adherence and compliance

## Abstract

Although direct‐acting antivirals (DAAs) for chronic hepatitis C virus (HCV) infection are highly efficacious and safe, treatment initiation is often limited in patients with neuropsychiatric disorders due to concerns over reduced treatment adherence and drug–drug interactions. Here, we report adherence, efficacy, safety and patient‐reported outcomes (PROs) from an integrated analysis of registrational studies using the pangenotypic DAA regimen of glecaprevir and pibrentasvir (G/P). Patients with chronic HCV genotypes 1‐6 infection with compensated liver disease (with or without cirrhosis) receiving G/P for 8, 12 or 16 weeks were included in this analysis. Patients were classified as having a psychiatric disorder based on medical history and/or co‐medications. Primary analyses assessed treatment adherence, efficacy (sustained virologic response at post‐treatment week 12; SVR12), safety and PROs. Among 2522 patients receiving G/P, 789 (31%) had a psychiatric disorder with the most common diagnoses being depression (64%; 506/789) and anxiety disorders (27%; 216/789). Treatment adherence was comparably high (>95%) in patients with and without psychiatric disorders. SVR12 rates were 97.3% (768/789; 95% CI = 96.2‐98.5) and 97.5% (1689/1733; 95% CI = 96.7‐98.2) in patients with and without psychiatric disorders, respectively. Among patients with psychiatric disorders, SVR12 rates remained >96% by individual psychiatric diagnoses and co‐medication classes. Overall, most adverse events (AEs) were mild‐to‐moderate in severity with serious AEs and AEs leading to G/P discontinuation occurring at similarly low rates in both patient populations. In conclusion, G/P treatment was highly efficacious, well‐tolerated and demonstrated high adherence rates in patients with chronic HCV infection and psychiatric disorders.

**Abbreviations**
AEs: adverse events
ALT: alanine aminotransferase
ATC: Anatomical Therapeutic Chemical
DAAs: direct‐acting antivirals
DDIs: drug–drug interactions
FSS: Fatigue Severity Scale
HCV: hepatitis C virus
HIV: human immunodeficiency virus
IFN: interferon
ITT: intent‐to‐treat
MCS: Mental Component Summary
PROs: patient‐reported outcomes
QoL: quality‐of‐life
RT‐PCR: reverse transcriptase‐polymerase chain reaction
SVR: sustained virologic response
TEAE: treatment‐emergent adverse event

## 1 INTRODUCTION

Chronic hepatitis C virus infection is associated with neuropsychiatric disorders in up to 50% of cases.^1^, ^2^ Historically, patients with comorbid psychiatric disorders were less likely to receive HCV treatment since interferon (IFN)‐based regimens can induce depression and other neuropsychiatric manifestations including insomnia, irritability and mood changes.^3^ Treatment adherence among this patient population was also a concern due to IFN's psychiatric side effect profile and perceived risks of lower adherence in patients with psychiatric and/or substance use disorders.^3^ Yet, fatigue and psychological issues contribute significantly to quality‐of‐life (QoL) impairments in patients with chronic HCV infection, both of which can be improved by the achievement of sustained virologic response (SVR).^4^, ^5^

The introduction of direct‐acting antivirals (DAAs) provided IFN‐free treatment regimens that likely are more suitable for patients with chronic HCV infection and comorbid psychiatric disorders. Both clinical trials and real‐world evidence have demonstrated that these all‐oral, IFN‐free regimens are highly efficacious and well tolerated with minimal treatment‐emergent neuropsychiatric side effects in patients with chronic HCV infection.^6^, ^7^, ^8^ Based on these data, recent HCV treatment guidelines recommend DAA regimens without any restrictions based on psychiatric comorbidities.^9^, ^10^ However, there has been more limited use of DAAs in patients with psychiatric disorders in both late phase trials and clinical practice potentially due to concerns about treatment adherence and drug–drug interactions (DDIs) with neuropsychiatric co‐medications.^3^, ^11^, ^12^, ^13^, ^14^ Thus, there is an unmet need to better understand the impact of psychiatric disorders on the treatment adherence, efficacy and safety of DAA regimens.

Glecaprevir (GLE; NS3/4A protease inhibitor identified by AbbVie and Enanta) and pibrentasvir (PIB; NS5A inhibitor) are potent pangenotypic inhibitors co‐formulated as G/P, an all‐oral, once‐daily and pangenotypic DAA regimen that demonstrated high efficacy, and favourable safety and DDI profiles in patients with chronic HCV infection. In vitro, glecaprevir and pibrentasvir exhibited nanomolar and picomolar potencies, respectively, against all major HCV genotypes and both retained their activity against most resistance‐associated substitutions.^15^, ^16^ Phase 1 trials investigated and thoroughly characterized the DDI profile of G/P, finding limited interactions and demonstrating that the majority of concomitant medications, including neuropsychiatric medications, can be safely taken with G/P without dose modification.^17^, ^18^ In late phase clinical trials, G/P was highly efficacious and safe in patients with chronic HCV genotypes 1‐6 infection including in patients with compensated cirrhosis, end‐stage renal disease and co‐infection with human immunodeficiency virus (HIV).^19^, ^20^, ^21^, ^22^, ^23^, ^24^, ^25^, ^26^ Preliminary reports from real‐world cohorts have supported these clinical trial findings with G/P showing similarly high effectiveness and a favourable safety profile in clinical practice.^27^, ^28^

Here, we present an integrated analysis of ten Phase 2 and Phase 3 studies aimed at evaluating the impact of psychiatric disorders on the treatment adherence, efficacy, safety and patient‐reported outcomes (PROs) with G/P treatment.

## 2 METHODS

### 2.1 Analysis set

Data were pooled from 2,522 patients with chronic HCV genotype 1‐6 infection and either without cirrhosis or with compensated cirrhosis who received G/P in ten Phase 2 and Phase 3 clinical trials that assessed efficacy, safety, treatment adherence and PROs (SURVEYOR‐I and SURVEYOR‐II and MAGELLAN‐1, and ENDURANCE‐1, ENDURANCE‐2, ENDURANCE‐3 and ENDURANCE‐4, and EXPEDITION‐1, EXPEDITION‐2 and EXPEDITION‐4).^19^, ^20^, ^21^, ^22^, ^23^, ^24^, ^25^, ^26^ This integrated analysis set included all patients who received at least one dose of glecaprevir 300 mg and pibrentasvir 120 mg either as separate tablets (Phase 2 formulation) or co‐formulated tablets dosed orally as three pills for a total 300mg/120mg dose (Phase 3 formulation). Both formulations were given as once‐daily, all‐oral regimens for 8, 12 or 16 weeks. All authors had access to data, and reviewed and approved the final manuscript.

### 2.2 Patients

Complete inclusion and exclusion criteria were similar across all clinical trials minus key trial‐specific eligibility criteria noted in Table S1. Adults (≥18 years of age) with chronic HCV genotype 1‐6 infection were eligible for the studies if they were positive for anti‐HCV antibody with a plasma HCV RNA viral load ≥10 000 IU/mL in Phase 2 trials or ≥1000 IU/mL in Phase 3 trials at the screening visit. Patients were eligible if they were either HCV treatment‐naïve or had prior treatment experience with IFN/pegylated (peg) IFN ± ribavirin (RBV) or sofosbuvir + RBV ± pegIFN. Medical history of psychiatric disorders was not exclusionary unless it was uncontrolled and made the subject an unsuitable candidate for the trial as assessed by the study investigator. Ongoing drug or alcohol use was not exclusionary unless it could preclude adherence as assessed by the study investigator. Other medical diagnoses were not exclusionary unless they were uncontrolled, including, but not limited to, cardiac, respiratory, gastrointestinal, haematologic, neurologic, or other medical diseases or disorders not related to existing HCV infection. Patients were excluded if they had active or suspected malignancy or history of malignancy in the past 5 years except basal cell skin cancer or cervical carcinoma in situ. Patients were also excluded if they required or could not safely discontinue the following medications: any herbal supplement, red yeast rice (monacolin K), St. John's Wort, carbamazepine, phenytoin, pentobarbital, phenobarbital, primidone, rifabutin, rifampin, atorvastatin, lovastatin, simvastatin, astemizole, cisapride and terfenadine. All patients provided written informed consent. Clinical trials were designed and conducted in accordance with the Good Clinical Practice guidelines, Declaration of Helsinki, and applicable local regulation and with approval from independent ethics committees or institutional review boards at all study sites.

In this post hoc analysis, patients receiving at least one dose of G/P were classified as having a psychiatric disorder by medical history and/or concomitant medication use. Medical history was used to classify patients as having a psychiatric disorder if they had been previously diagnosed with any of the following psychiatric or neurological disorders including anxiety, bipolar disorder, cognitive or psychiatric disorder, depression, Parkinson's disease, and seizure disorder or convulsions. Concomitant medication use was used to classify patients as having a psychiatric disorder if patients were receiving antidepressants or antipsychotics as defined by the Anatomical Therapeutic Chemical (ATC) Classification System.

### 2.3 Procedures

Real‐time reverse transcriptase‐polymerase chain reaction (RT‐PCR) was utilized to quantify plasma HCV RNA for both baseline viral load and SVR12 assessments; assay details are described in the Supporting Information. HCV genotype was determined using the Versant® HCV Genotype Inno LiPA Assay, Version 2.0 or higher (LiPA; Siemens Healthcare Diagnostics, Tarrytown, NY) and confirmed by phylogenetic analysis of viral sequences. Treatment adherence was assessed using pill count at visits every 4 weeks during the treatment period including at the end of treatment visit. Adherence was defined as the lowest adherence to G/P being ≥80% and ≤120% at all intervals between baseline and end of treatment. Post hoc analysis imputed any missing values for drug adherence at any of the treatment visits as the lowest obtained value from the patient's other visits. An exploratory analysis assessed PROs related to neuropsychiatric function for patients with or without psychiatric disorders by evaluating the mean change from baseline to post‐treatment week 12 on the Short‐Form 36 (SF‐36) Mental Component Summary (MCS) and Fatigue Severity Scale (FSS).

Safety was evaluated by monitoring adverse events (AEs), vital signs, physical examination findings, electrocardiography and clinical laboratory tests. Nonserious and serious AEs were monitored throughout G/P treatment until 30 days post‐treatment and up to 24 weeks post‐treatment, respectively. Any AE with an onset date after the first G/P dose and no more than 30 days after the last G/P dose was classified as a treatment‐emergent adverse event (TEAE). All AEs were coded using the Medical Dictionary for Regulatory Activities (MedDRA) and were assessed for their relationship with G/P by study investigators.

### 2.4 Endpoints

The primary endpoints of this integrated analysis were the efficacy and safety of G/P in patients with chronic HCV genotype 1‐6 infection with or without a psychiatric disorder. Efficacy was evaluated by SVR (HCV RNA < lower limit of quantification) at 12 weeks post‐treatment in the intent‐to‐treat (ITT) population for patients with or without a psychiatric disorder. Safety was evaluated by characterizing reported AEs and the number and percentage of G/P‐treated patients who reported treatment‐emergent adverse events and laboratory abnormalities, both in total and stratified by the presence or absence of a psychiatric disorder. Additional endpoints included analyses of treatment adherence, patient‐reported health outcomes related to neurocognitive function and subgroup analyses of SVR12 in patients by neuropsychiatric co‐medications or psychiatric diagnoses.

### 2.5 Statistical analysis

The number and percentage of patients in the ITT population achieving SVR12 for patients with or without psychiatric disorders were summarized with two‐sided 95% confidence intervals calculated using the normal approximation to the binomial distribution. Further analyses evaluated SVR12 rates by ITT analysis in subgroups of patients with psychiatric disorders in order to assess whether common psychiatric disorders and/or co‐medications affected achievement of SVR12.

## 3 RESULTS

### 3.1 Baseline patient demographics and characteristics

This analysis of Phase 2 and Phase 3 clinical trial data consisted of 2522 patients with chronic HCV genotypes 1‐6 infection treated with G/P for either 8, 12 or 16 weeks following enrolment between 7 October 2014 and 12 September 2016. Overall, this clinical trial population included 789 (31%) patients classified as having a psychiatric disorder based on a previous medical history of ≥1 psychiatric disorder (90%; 708/789) and/or concomitant psychiatric medication use (58%; 455/789). Table 1 delineates the baseline and disease characteristics of patients with or without a psychiatric disorder. Patients with psychiatric disorders were more often female (49% vs 40%, respectively), white (87% vs 77%, respectively), GT3‐infected (32% vs 24%, respectively) and had higher prevalence of cirrhosis (16% vs 11%, respectively) and medical history of injection drug use (56% vs 34%, respectively) compared to those without a psychiatric disorder.

**Table 1:** Baseline demographics and disease characteristics for patients with or without a psychiatric disorder

Characteristic	Patients with psychiatric disorders N = 789	Patients without a psychiatric disorder N = 1733
Male, n (%)	403 (51)	1043 (60)
Age, median (range), years	53 (21‐82)	54 (19‐88)
Race, n (%)		
White	685 (87)	1,334 (77)
Black or African‐American	53 (7)	121 (7)
Asian	36 (5)	242 (14)
Other	13 (2)	35 (2)
Missing	2	1
BMI, median (range), kg/m^2^	26.4 (17.3‐55.4)	25.6 (17.4‐65.7)
Baseline HCV RNA level, median (range), log_10_ IU/mL	6.3 (1.2‐7.6)	6.2 (0.7‐7.8)
HCV genotype, n (%)		
GT1	331 (42)	764 (44)
GT2	144 (18)	332 (19)
GT3	251 (32)	418 (24)
GT4‐6	63 (8)	219 (13)
HCV treatment‐naïve, n (%)	568 (72)	1,197 (69)
HCV treatment‐experienced, n (%)	221 (28)	536 (31)
IFN‐experienced	180 (23)	464 (27)
PI/NS5A‐experienced	41 (5)	72 (4)
Fibrosis status, n (%)		
F0‐F1	541 (69)	1,230 (71)
F2	41 (5)	126 (7)
F3	83 (11)	177 (10)
F4	123 (16)	196 (11)
Missing	1	4
G/P treatment duration, n (%)		
8 wk	312 (40)	653 (38)
12 wk	433 (55)	1004 (58)
16 wk	44 (6)	76 (4)
History of injection drug use^a^	439 (56)	595 (34)
History of psychiatric disorders ≥5% of patients, n (%)		
Depression	506 (64)	N/A^b^
Anxiety	216 (27)	N/A^b^
Cognitive or psychiatric Disorder	97 (12)	N/A^b^
Bipolar disorder	57 (7)	N/A^b^
Concomitant neuropsychiatric drug use in ≥10% of patients by class, n (%)^c^		
Antidepressants	396 (50)	N/A^b^
Opioids	272 (34)	221 (13)
Anxiolytics	244 (31)	74 (4)
Antiepileptic	217 (28)	69 (4)
Hypnotics and sedatives	159 (20)	98 (6)
Antipsychotics	117 (15)	N/A^b^
Drugs used for substance use disorders^d^	116 (15)	98 (6)
Treatment adherent, n (%)	753 (95)	1676 (97)

Abbreviations: BMI, body mass index; HCV, hepatitis C virus; GT, genotype; IFN, interferon; PI, protease inhibitor; N/A, not applicable.

^a^ Includes all patients who previously injected drugs regardless of how recent the patient‐injected drugs.

^b^ Not applicable to patients without psychiatric disorders since this parameter was used to define the population with psychiatric disorders.

^c^ Concomitant medications grouped by Anatomical Therapeutic Chemical (ATC) Classification System.

^d^ Includes the following drugs: methadone, buprenorphine (with or without naloxone), nicotine, diamorphine, levomethadone, disulfiram, naltrexone, varenicline, acamprosate and naloxone.

The most common concomitant medication for each of these neuropsychiatric drug classes is listed in Table 2 for patients with or without psychiatric disorders when appropriate. Among the most common neuropsychiatric medications used concomitantly with G/P, the University of Liverpool website only identified quetiapine (N = 47), hydrocodone (N = 77) and oxycodone (N = 81) as neuropsychiatric co‐medications with potential DDIs with G/P (Table 2).

**Table 2:** Most common neuropsychiatric co‐medications by anatomical therapeutic chemical (ATC) class

Drug class	Medication	Patients with Psychiatric Disorders N = 789 (n, %)	Patients without a Psychiatric Disorder N = 1733 (n, %)	Overall N = 2522 (n, %)
Antidepressants^a^	Trazodone	62 (7.9)	0	62 (2.5)
	Escitalopram	57 (7.3)	0	57 (2.3)
	Citalopram	48 (6.1)	0	48 (1.9)
	Bupropion	45 (5.7)	0	45 (1.8)
	Sertraline	45 (5.7)	0	45 (1.8)
Opioids	Codeine^b^	49 (6.2)	46 (2.7)	95 (3.8)
	Tramadol	45 (5.7)	36 (2.1)	81 (3.2)
	Oxycodone^b^, ^c^	52 (6.6)	29 (1.7)	81 (3.2)
	Hydrocodone^b^, ^c^	50 (6.3)	27 (1.6)	77 (3.1)
	Morphine	17 (2.2)	12 (0.7)	29 (1.1)
Anxiolytics	Alprazolam	65 (8.2)	21 (1.2)	86 (3.4)
	Clonazepam	44 (5.6)	8 (0.5)	52 (2.1)
	Diazepam	42 (5.3)	10 (0.6)	52 (2.1)
	Lorazepam	40 (5.1)	9 (0.5)	49 (1.9)
	Hydroxyzine	15 (1.9)	14 (0.8)	29 (1.1)
Antiepileptic	Gabapentin	68 (8.6)	31 (1.8)	99 (3.9)
	Pregabalin	21 (2.7)	8 (0.5)	29 (1.1)
	Lamotrigine	18 (2.3)	1 (<0.1)	19 (0.8)
	Levetiracetam	15 (1.9)	2 (0.1)	17 (0.7)
	Valproic Acid	7 (0.9)	2 (0.1)	9 (0.4)
Hypnotics and sedatives	Zolpidem	43 (5.4)	24 (1.4)	67 (2.4)
	Diphenhydramine	27 (3.4)	17 (1)	44 (1.7)
	Zopiclone	20 (2.5)	20 (1.2)	40 (1.6)
	Melatonin	20 (2.5)	9 (0.5)	29 (1.1)
	Promethazine	17 (2.2)	5 (0.3)	22 (0.9)
Antipsychotics^a^	Quetiapine^c^	47 (6)	0	47 (1.9)
	Risperidone	18 (2.3)	0	18 (0.7)
	Lithium	11 (1.4)	0	11 (0.4)
	Olanzapine	11 (1.4)	0	11 (0.4)
	Aripiprazole	9 (1.1)	0	9 (0.4)
Drugs used in substance use disorders	Methadone	60 (7.6)	60 (3.5)	120 (4.8)
	Buprenorphine with naloxone	19 (2.4)	13 (0.8)	32 (1.3)
	Buprenorphine	18 (2.3)	8 (0.5)	26 (1.0)
	Nicotine	10 (1.3)	14 (0.8)	24 (1.0)
	Diamorphine	3 (0.4)	2 (0.1)	5 (0.2)

Note

Anatomical Therapeutic Chemical.

^a^ Not applicable to patients without psychiatric disorders since patients taking these co‐medications were defined as having a psychiatric disorder.

^b^ Includes patients taking a regimen containing the listed generic drug name.

^c^ Medications with potential interactions with G/P based on the University of Liverpool website (http://www.hep-druginteractions.org).

### 3.2 Treatment adherence

Treatment adherence was similarly high among patients with or without a psychiatric disorder (95.4% and 96.7%, respectively; Table 1). Table 3 illustrates high (>94%) treatment adherence rates among patients with histories of depression or anxiety disorders; treatment adherence was lowest in patients with a history of bipolar disorder (89.5%). Treatment adherence remained high (>94%) for those prescribed neuropsychiatric co‐medication classes (Table 3) and for patients taking >1 psychiatric co‐medication or 16‐week G/P treatment (Table S2).

**Table 3:** Treatment adherence in patients with psychiatric disorder by diagnosis and neuropsychiatric co‐medication

Characteristic, % (n/N)	G/P treatment adherence
History of Psychiatric Disorder^a^	
Depression	95.7 (484/506)
Anxiety	95.4 (206/216)
Bipolar disorder	89.5 (51/57)
Concomitant Neuropsychiatric drug use	
Antidepressants	95.2 (377/396)
Opioids	94.9 (258/272)
Anxiolytics	95.1 (232/244)
Antiepileptics	94.5 (205/217)
Hypnotics and sedatives	96.2 (153/159)
Antipsychotics	94.9 (111/117)
Drugs used in substance use disorders	96.6 (112/116)

Abbreviation: G/P, glecaprevir/pibrentasvir.

^a^ Reported in previous patient medical history.

### 3.3 Efficacy outcomes

**Figure 1:**
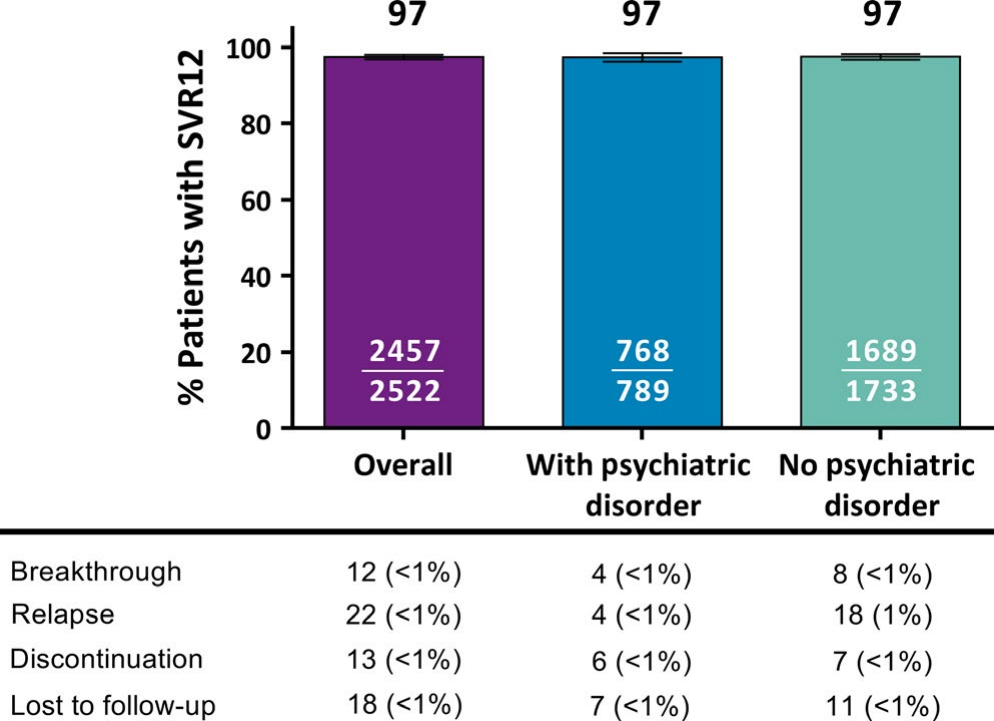
SVR12 by ITT analysis for patients with and without a psychiatric disorder. G/P efficacy, defined as SVR12, is reported both overall and by presence or absence of psychiatric disorders. Reasons for nonresponse are reported for virologic (breakthrough or relapse) and nonvirologic (discontinuation or lost to follow‐up) failure

SVR12 rates for the ITT population were 97.3% (768/789; 95% CI = 96.2‐98.5) in patients with a psychiatric disorder compared to 97.5% (1689/1733; 95% CI = 96.7‐98.2) in those without a psychiatric disorder (Figure 1). The rate of virologic failure was 1.0% (8/789) among patients with a psychiatric disorder compared to 1.5% (26/1733) in patients without a psychiatric disorder. Nonvirologic failures from premature discontinuations of G/P and lost to follow‐up each occurred in less than 1% of patients regardless of the presence or absence of a psychiatric disorder.

Subgroup analyses in the ITT population demonstrated numerically comparable SVR12 rates in patients with and without a psychiatric disorder regardless of patient characteristics including age, fibrosis stage, treatment duration and treatment adherence (Figures S1 and S2). Although SVR12 rates by ITT analysis were numerically lower in all patients who were nonadherent than in those who were adherent, exclusion of nonvirologic failures (early discontinuations or lost to follow‐up) using a modified ITT analysis demonstrated numerically similar SVR12 rates regardless of treatment adherence (Figure S2). SVR12 rates (%, n/N) remained high in all patients taking a neuropsychiatric co‐medication with a potential DDI, namely quetiapine (100%, 47/47), oxycodone (93.8%, 76/81) and hydrocodone (97.4%, 75/77) (Figure S3).

**Figure 2:**
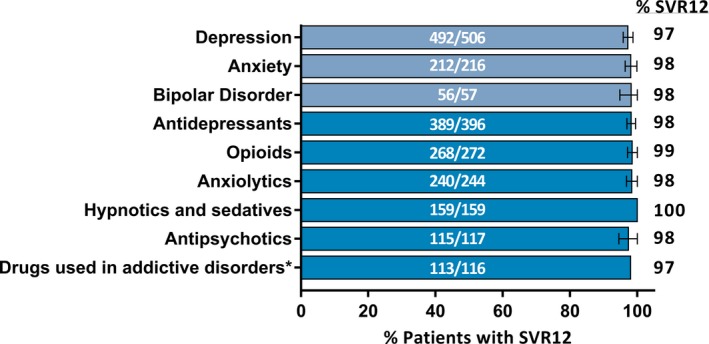
SVR12 by ITT analysis stratified by psychiatric diagnosis or neuropsychiatric co‐medication for patients with psychiatric disorders. SVR12 data in patients with psychiatric disorders further stratified by individual psychiatric disorders (light blue) or neuropsychiatric co‐medications (blue)

SVR12 rates by ITT analysis were further stratified for patients with psychiatric disorders by common psychiatric diagnoses and neuropsychiatric co‐medication classes (Figure 2). SVR12 rates were >97% for patients with the most common psychiatric disorders, namely depression (97.2%; 95% CI = 95.8‐98.7), anxiety disorders (98.1%; 95% CI = 96.4‐99.9) and bipolar disorder (98.2%; 95% CI = 94.8‐100). Similarly, SVR12 rates were >97% for all of the most common neuropsychiatric co‐medication classes (Figure 2) and remained high (>96%) for patients with psychiatric disorders not taking a concomitant psychiatric medication (Figure S4).

### 3.4 Safety outcomes

Among the 1697 (67%) patients experiencing an AE, 610 (77%) patients with a psychiatric disorder reported an AE compared to 1087 (63%) patients without a psychiatric disorder (Table 4). Most AEs were classified as either mild (Grade 1) or moderate (Grade 2) in severity for patients with (568/610; 93%) and without a psychiatric disorder (1035/1087; 95%). Overall, the most common AEs occurring in ≥10% of patients with or without a psychiatric disorder, respectively, were headache (20% vs 16%), fatigue (18% vs 13%) and nausea (13% vs 8%), and these tended to occur more often in patients with a psychiatric disorder. Neuropsychiatric AEs were not observed in ≥10% of patients for either population, but tended to occur more often in patients with a psychiatric disorder than those without a psychiatric disorder (8% vs 3%, respectively).

**Table 4:** Adverse events and laboratory abnormalities

Event, n (%)	Patients with psychiatric disorder N = 789	Patients without a psychiatric disorder N = 1733
Any AE	610 (77)	1087 (63)
Any neuropsychiatric AE^a^	64 (8)	52 (3)
Serious AE	30 (4)	47 (3)
DAA‐related serious AE	0	1 (<1)^b^
AEs leading to discontinuation	5 (<1)	8 (<1)
DAA‐related AEs leading to discontinuation	2 (<1)^c^	3 (<1)^d^
AEs occurring in ≥10% of patients		
Headache	158 (20)	273 (16)
Fatigue	140 (18)	223 (13)
Nausea	102 (13)	131 (8)
Laboratory Abnormalities		
ALT, grade ≥3^e^	1 (<1)	1 (<1)
AST, grade ≥3	3 (<1)	3 (<1)
Total bilirubin, grade ≥3	6 (<1)	4 (<1)

Abbreviations: AE, adverse event; ALT, alanine aminotransferase; AST, aspartate aminotransferase; DAA, direct‐acting antiviral.

^a^ Any AE included under standardized MedDRA queries of depression, suicide/self‐injury and psychosis.

^b^ Grade 3 transient ischaemic attacks on Day 11 in patient with history of smoking, obesity and a cardiac conduction abnormality along with elevated haemoglobin and haematocrit at screening. This patient subsequently experienced another SAE of transient ischaemic attack on Day 36 (24 d after discontinuing G/P treatment).

^c^ Two patients experienced 8 nonserious AEs leading to treatment discontinuation (dyspepsia, nausea, diarrhoea, dizziness, fatigue, malaise, abdominal pain and headache).

^d^ One patient experienced a nonserious AE of diarrhoea on Day 27; two other patients with pre‐existing conditions experienced serious AEs of transient ischaemic attack on Day 11 and pruritus on Day 61.

^e^ Post‐nadir increase (in grade).

Adverse events leading to G/P discontinuation and G/P‐related serious AEs were rare (<1%) in both patients with and without a psychiatric disorder (Table 4). Serious AEs occurred at similar frequencies in patients with and without psychiatric disorders. No patients with psychiatric disorders experienced a G/P‐related serious AE as assessed by the study investigator. G/P‐related AEs leading to discontinuation were reported in 5 (<1%) patients, including two patients with psychiatric disorders who discontinued due to nonserious AEs of dyspepsia on Day 70 and adverse events (nausea, diarrhoea, dizziness, fatigue, malaise, abdominal pain and headache) at Day 5, respectively. Similar rates of AEs and G/P‐related AEs leading to discontinuation were observed in patients taking a neuropsychiatric co‐medication with a potential DDI, namely quetiapine, oxycodone and hydrocodone (Table S3). Although serious AEs were reported at numerically higher rates for patients taking oxycodone (11%) or hydrocodone (6%), none of the serious AEs were due to respiratory depression or related to G/P (Table S3).

Laboratory abnormalities were also rare (<1%) in patients with and without a psychiatric disorder (Table 4). Grade 3 elevations in alanine aminotransferase (ALT) were rare (<1%) in both populations, with 1 (<1%) patient with a psychiatric disorder exhibiting a single Grade 3 ALT elevation at Day 7 from a previous Grade 2 ALT elevation. The Grade 3 ALT elevation in the patient without a psychiatric disorder occurred in the context of multiple gallstones. Most Grade 3 elevations in total bilirubin occurred in patients with pre‐existing elevations (Grade 1 or 2) and were transient in nature, resulting predominantly from increased indirect bilirubin fraction consistent with known glecaprevir effects on bilirubin transport (inhibition of OATP1B1) and conjugation.^18^

### 3.5 Exploratory analysis of patient‐reported outcomes

Mean change (±SD) in PROs related to neuropsychiatric function was evaluated to assess QoL changes in patients with and without psychiatric disorders using the SF‐36 MCS and FSS from 8 of the 10 clinical trials (SURVEYOR‐I and SURVEYOR‐II, ENDURANCE‐2, ENDURANCE‐3 and ENDURANCE‐4, and EXPEDITION‐1, EXPEDITION‐2 and EXPEDITION‐4). With available data from the SF‐36 MCS for 538 patients with a psychiatric disorder and 1077 patients without a psychiatric disorder, there was a trend towards increased mental health at post‐treatment week 12. A numerically higher increase was observed in patients with a psychiatric disorder (3.6 ± 11.7) compared to those without a psychiatric disorder (2.2 ± 9.6). Likewise, available data from 542 and 1080 patients with and without psychiatric disorders, respectively, showed a trend at post‐treatment week 12 towards slightly decreased fatigue scores as assessed by the FSS in patients with and without psychiatric disorders (−0.5 ± 1.6 and −0.3 ± 1.6, respectively).

## 4 DISCUSSION

The pangenotypic, once‐daily DAA regimen of G/P achieved high (>97%) SVR12 rates in both patients with and without a psychiatric disorder. In patients with a psychiatric disorder, G/P achieved >96% SVR12 rates regardless of psychiatric diagnoses or the prescription of neuropsychiatric co‐medications, in spite of a numerically higher prevalence of GT3 infection in patients with psychiatric disorders likely due to the higher prevalence of GT3 infection among patients with a history of substance use.^29^, ^30^ Overall, treatment adherence was >95% for both patients with or without psychiatric disorders. G/P was well tolerated in both patients with and without psychiatric disorders, exhibiting similarly low rates of serious AEs and AEs leading to G/P discontinuation. Patients with psychiatric disorders treated with G/P did not have higher rates of lost to follow‐up or premature discontinuations resulting in non‐SVR compared to patients without psychiatric disorders. An exploratory analysis of PROs related to neuropsychiatric function demonstrated trends towards improvements in mental health and fatigue especially in patients with psychiatric disorders. Overall, G/P is highly efficacious and safe with high adherence rates in this large clinical trial cohort of patients with psychiatric disorders. Our findings are consistent with those of other DAA regimens in clinical trial populations.^31^, ^32^

Adherence among patients with chronic HCV infection and comorbid psychiatric disorders remains a concern despite the advent of IFN‐free DAA treatments. Lower adherence is hypothesized due to cases of severe, untreated psychiatric disorders and higher rates of comorbid substance use disorders 3, 11, 12, 13; however, DAA regimens have shown similarly high adherence rates in clinical trials among patient populations traditionally considered to be at‐risk for nonadherence including patients with psychiatric disorders, people who inject drugs and patients on stable opioid substitution therapy.^29^, ^31^ In its clinical trial programme, overall adherence to G/P regimens has been reported at >95% with comparable rates among patients with psychiatric disorders, people who inject drugs and patients on stable opioid substitution therapy.^33^, ^34^ Although treatment adherence was almost 90% in patients with bipolar disorder, nonadherence to medication was more common among patients with bipolar disorder in line with previous studies.^35^ However, it is conceivable that real‐world cohorts with more severe, untreated psychiatric disorders may be at‐risk for lower treatment adherence with DAAs including G/P. Shorter treatment regimens with G/P may facilitate improved adherence in clinical practice since other DAA regimens have shown decreased adherence over 12‐week treatment.^36^ Additionally, as evidenced by the low rate of virologic failure using a modified ITT analysis, G/P provides a durable treatment regimen with similarly high efficacy in patients who are nonadherent (<80%) compared to those who are adherent.^37^

In the era of IFN‐free DAAs, concerns over DDIs, not safety, are relevant to treatment decisions for patients with psychiatric disorders. Similar to other DAAs, these data generated with G/P regimens further support the consistent finding that DAAs are well‐tolerated and are not associated with increased psychiatric AEs as seen with IFN‐based treatments.^3^, ^31^, ^38^, ^39^ Although treatment‐emergent psychiatric AEs are not a concern with DAAs, current treatment guidelines recommend assessment for potential DDIs prior to HCV treatment initiation.^9^, ^10^ This is particularly relevant for patients taking neuropsychiatric medications since all available DAAs, including G/P, are contraindicated with carbamazepine and most other anticonvulsants due to known DDIs.^18^, ^40^, ^41^, ^42^, ^43^ Among other common co‐medications including drugs for neuropsychiatric disorders, G/P demonstrated limited potential DDIs; however, its activity as a weak CYP3A4 inhibitor could potentiate DDIs with the common neuropsychiatric co‐medications, quetiapine, hydrocodone and oxycodone.^18^ Overall, G/P was safe and efficacious in patients taking any of these neuropsychiatric co‐medications with potential DDIs.

Neuropsychiatric manifestations of HCV are commonly reported, and their severity can be alleviated with the achievement of SVR.^1^ In G/P's clinical trials programme reported here, the trends towards improvements in PROs related to neuropsychiatric function mirror those improvements reported with other DAA regimens, particularly the numerically larger effect in patients with psychiatric disorders; thus, these findings warrant further investigation.^4^, ^39^, ^44^, ^45^, ^46^, ^47^, ^48^, ^49^ Further long‐term follow‐up may be necessary in order to observe whether these trends are both robust and durable over time.

There are limitations to this analysis that are inherent to its design. Since this is a post hoc analysis, the comparisons between patients with and without a psychiatric disorder were not pre‐specified and, thus, are not powered to assess for differences between these patient populations. It is not valid to compare psychiatric AEs between patients with or without a psychiatric disorder as there are most likely to be different risk factors or comorbidities for each of these groups contributing to their psychiatric AEs. The ideal analysis would involve a direct comparison of patients treated with or without G/P with psychiatric disorders. Additionally, the patients with psychiatric disorders enrolled in clinical trials are a more selected population potentially with more controlled disorders and an underrepresentation of Black or African‐Americans compared to patients in the real‐world setting; therefore, it is important to validate these findings with data obtained from real‐world clinical practice. Of note, 30% of patients with psychiatric disorders were treatment‐experienced with most receiving interferon‐containing regimens, suggesting that these patients had less severe psychiatric disorders since interferon is contraindicated for patients with severe psychiatric disorders. Additionally, 77 patients were excluded from these studies as a result of a clinically significant abnormality other than HCV infection due to a cluster of clinical abnormalities of which may or may not have included an uncontrolled psychiatric disorder. Full details are listed in the Supporting Information. Finally, treatment adherence was assessed in these clinical trials by measuring pill counts that provide quantitative data on adherence at specified visits, but can overestimate overall adherence and lacks any information on dose timing.^50^

Overall, among the 789 (31%) patients with a psychiatric disorder in its clinical trials programme, G/P demonstrated comparably high SVR12 rates and treatment adherence to those observed in patients without a psychiatric disorder. Despite a higher rate of mild‐to‐moderate AEs in patients with psychiatric disorders, G/P was well tolerated with similarly low rates of serious AEs or AEs leading to G/P discontinuation in patients with or without psychiatric disorders. Thus, G/P is a pangenotypic regimen suitable for treatment of the ≤50% of patients with chronic HCV infection and comorbid psychiatric disorders.

## CONFLICT OF INTERESTS

David Back: Advisory board member/speakers bureau and receives honorarium from: Gilead, Merck, AbbVie, Bristol‐Myers Squibb, Janssen; received research grant funding from: Gilead, Merck, AbbVie, Bristol‐Myers Squibb, Janssen; received travel sponsorship from: AbbVie. Pamela Belperio: Nothing to disclose. Fiona Marra: consulting or grants from AbbVie, Gilead, Merck, Janssen and Viiv. Francesco Negro: advisor to Gilead, AbbVie, Merck. Unrestricted research grant from AbbVie. Investigator initiated study supported by Gilead. Andrew Talal: Research grants: AbbVie, Merck, Gilead, Intercept, Conatus, Abbott, Genfit, Center for AIDS Research, Patient‐Centered Outcomes Research Institute (PCORI); Advisor: AbbVie, Merck, Abbott Diagnostics, Chronic Liver Disease Foundation; Speaker's Bureau: Chronic Liver Disease Foundation. Mark Bondin, Caroline Park, ZhenZhen Zhang, Brett Pinsky, Federico Mensa, Eric Crown are employees of AbbVie, Inc and may hold stock or stock options.
