# Supplementary material for: Efficacy and safety of glecaprevir/pibrentasvir in patients with chronic HCV infection and psychiatric disorders: An integrated analysis
Source: J Viral Hepat. 2019 May 20;26(8):951–60. doi: 10.1111/jvh.13110 (PMC6852431; doi:10.1111/jvh.13110)
Supplement: Supplementary file 1 [file JVH-26-951-s001.docx]

# Online Supplemental Information

# Efficacy and Safety of Glecaprevir/Pibrentasvir in Patients with Chronic HCV Infection and Psychiatric Disorders: An Integrated Analysis

David Back, Pamela Belperio, Mark Bondin, Francesco Negro, Andrew H. Talal, Caroline Park, ZhenZhen Zhang, Brett Pinsky, Eric Crown, Federico J. Mensa, Fiona Marra

# Table of Contents

HCV RNA Assays………………………………………………………………………………...……..3

[Eligibility Criteria 4](#_Toc479596269)

[Inclusion 4](#_Toc479596270)

Supporting Table 1. Trial-Specific Inclusion Criteria………………………...…...……………….…7

[Exclusion 7](#_Toc479596270)

Supporting Table 2. Treatment Compliance by Number of Psychiatric Co-medications and Treatment Duration ………………………………………………………………. ………………..…………..…11

[Supporting Figure 1. Subgroup Efficacy Analysis by Baseline Characteristics in Patients with or without Psychiatric Disorder. 12](#_Toc479596272)

[Supporting Figure 2. Efficacy by ITT and modified ITT analysis for Patients with or without Psychiatric Disorders by Adherence. 13](#_Toc479596272)

[Supporting Figure 3. Efficacy of G/P in Patients taking a Neuropsychiatric Co-Medication with a Potential DDI by ITT analysis. ..14](#_Toc479596272)

[Supporting Figure 4. Efficacy by ITT Analysis in Patients by Number of Psychiatric Co-medications Taken in Patients with Psychiatric Disorders. 15](#_Toc479596272)

Supporting Table 3. Adverse Events in Patients taking a Neuropsychiatric Co-Medication with a Potential DDI. ..16

# HCV RNA Assays

For the 201 patients enrolled in Phase 2 trials as well as 203 patients enrolled in the Phase 3 SURVEYOR-II Part 4 study, the High Pure System was used to manually prepare each sample followed by central laboratory testing for plasma HCV RNA levels using the COBAS TaqMan® real-time reverse transcriptase-polymerase chain reaction (RT-PCR) assay v. 2.0 (Roche Molecular Diagnostics, Pleasanton, CA), which has a LLOQ of 25 IU/mL, regardless of genotype. The LLOD is 15.0 for genotypes 1 and 3, and 5.6, 12.0, 3.7, and 20.4 IU/mL for HCV genotype 2, 4, 5, and 6, respectively. For patients enrolled in Phase 3 trials (N=2118, excluding the 203 enrolled in SURVEYOR-II Part 4), the central laboratory used the COBAS Ampliprep/TaqMan® real-time RT-PCR assay v. 2.0 (Roche Molecular Diagnostics, Pleasanton, CA), which has a lower limit of quantification (LLOQ) and a lower limit of detection (LLOD) of 15 IU/mL regardless of genotype, to quantify plasma HCV RNA levels for each sample collected,.

# Eligibility Criteria

## Inclusion

1. Male or female, at least 18 years of age at time of Screening with an upper limit of 70 years of age only in Phase 2 trials.
2. If female, subject must be either:
   1. Practicing one effective method of birth control with male partner(s) from screening to 30 days after stopping study drug
   2. Postmenopausal for at least 2 years prior to screening
   3. Or permanently surgically sterile (defined as bilateral tubal ligation, bilateral oophorectomy, or hysterectomy) or has a vasectomized partner(s)
3. Females of childbearing potential must have a negative serum pregnancy test result at Screening, and a negative urine pregnancy test at Study Day 1.

Females of non-childbearing potential (either postmenopausal or permanently surgically sterile) at Screening do not require pregnancy testing.

1. Sexually active males must be surgically sterile or have male partners only, or if sexually active with female partner(s) of childbearing potential must agree to practice at least one effective form of birth control
2. Screening laboratory result indicating HCV GT1-, 2-, 3-, 4-, 5-, or 6-infection; infection with more than one genotype was not permitted.
3. Subject has positive anti-HCV Ab and plasma HCV RNA viral load ≥10,000 for Phase 2 trials and ≥1000 IU/mL for Phase 3 trials at Screening Visit.
4. Chronic HCV infection defined as one of the following:
   1. Positive for anti-HCV antibody (Ab) or HCV RNA at least 6 months before Screening; or
   2. A liver biopsy consistent with chronic HCV infection; or
   3. Abnormal alanine aminotransferase (ALT) levels for at least 6 months before Screening (only used in Phase 3 trials).
5. Subject must be HCV treatment-naïve (i.e., subject has not received a single dose of any approved or investigational anti-HCV medication) or HCV treatment-experienced (subject has failed prior IFN or pegIFN with or without RBV or SOF plus RBV with or without pegIFN). Previous HCV treatment must have been completed ≥ 2 months prior to Screening.
6. Body Mass Index (BMI) is ≥ 18.0 kg/m^2^ at the time of Screening (with an upper limit of 38.0 kg/m^2^ in Phase 2 studies). BMI is calculated as weight measured in kilograms (kg) divided by the square of height measured in meters (m).
7. Subject must voluntarily sign and date an informed consent form, approved by an Institutional Review Board (IRB)/Independent Ethics Committee (IEC) prior to the initiation of any Screening or study specific procedures.
8. Subjects must be able to understand and adhere to the study visit schedule and all other protocol requirements.
9. Subject must be documented as non-cirrhotic or cirrhotic defined as meeting one of the following criteria:

**Non-Cirrhotics**

- 1. A liver biopsy within 24 months prior to or during Screening demonstrating the absence of cirrhosis, e.g., a METAVIR, Batts-Ludwig, Knodell, IASL, Scheuer, or Laennec fibrosis score of ≤ 3, Ishak fibrosis score of ≤ 4; or
  2. A FibroScan® score of < 12.5 kPa within ≤ 6 months of Screening or during Screening period (FibroScan® must be approved by the local regulatory agency to qualify for entrance criteria); or
     1. Subjects with indeterminate FibroScan® score (12.5≤ score < 14.6), must have a qualifying liver biopsy
  3. A Screening FibroTest score of ≤ 0.48 and Aspartate Aminotransferase to Platelet Ratio Index (APRI) < 1.
     1. Subjects with indeterminate Fibrotest (0.48< result <0.75), or conflicting FibroTest and APRI results (e.g., FibroTest ≤ 0.48, but APRI ≥ 1) must have a qualifying liver FibroScan® or biopsy.

**Cirrhotic**

- - Previous histologic diagnosis of cirrhosis on liver biopsy, e.g., METAVIR, Batts-Ludwig, Knodell, IASL, Scheuer, or Laennec fibrosis score of > 3, Ishak score of > 4 or on a liver biopsy conducted during Screening; or
  - A FibroScan® score of ≥12.5 kPa within ≤ 6 months of Screening or during Screening period (FibroScan® must be approved by the local regulatory agency to qualify for entrance criteria); or
  - A Screening FibroTest result that is ≥0.75 and an APRI > 2.
    1. In the absence of a definitive diagnosis of presence or absence of cirrhosis by Fibrotest/APRI using the above criteria (indeterminate FibroTest [0.48 < result < 0.75], or conflicting FibroTest and APRI results [e.g., FibroTest ≤ 0.48, but APRI ≥ 1]), a liver biopsy or FibroScan® is required. Liver biopsy results will supersede Fibrotest/APRI or FibroScan® results and be considered definitive.
    2. FibroScan® results will supersede Fibrotest/APRI results for the determination of presence or absence of cirrhosis

1. Cirrhotic Subjects Only: Compensated cirrhosis defined as Child-Pugh score of ≤ 6 at Screening and no current or past evidence of Child-Pugh B or C Classification or clinical history of liver decompensation including ascites noted on physical exam, hepatic encephalopathy or esophageal variceal bleeding.
2. Cirrhotic Subjects Only: Absence of hepatocellular carcinoma (HCC) as indicated by a negative ultrasound, computed tomography (CT) scan or magnetic resonance imaging (MRI) within 3 months prior to Screening or a negative ultrasound at Screening. Subjects who have an ultrasound with results suspicious of HCC followed by a subsequent negative CT or MRI of the liver will be eligible for the study.

## Supporting Table 1: Trial-Specific Inclusion Criteria

| Study | Genotypes (GT) | Treatment Duration | Population included |
| --- | --- | --- | --- |
| SURVEYOR-I | GT1, 4-6 | 8 weeks  12 weeks | Treatment naïve/PRS-experienced  ± Compensated cirrhosis |
| SURVEYOR-II | GT2-6 | 8 weeks  12 weeks  16 weeks | Treatment naïve/PRS-experienced  ± Compensated cirrhosis (GT3) |
| MAGELLAN-I | GT1, 4-6 | 12 weeks  16 weeks | DAA-experienced  ± Compensated cirrhosis |
| ENDURANCE-1 | GT1 | 8 weeks  12 weeks | HIV-1 co-infection  + Treatment naïve/PRS-experienced |
| ENDURANCE-2 | GT2 | 12 weeks | Treatment naïve/PRS-experienced |
| ENDURANCE-3 | GT3 | 8 weeks  12 weeks | Treatment-naïve |
| ENDURANCE-4 | GT4-6 | 12 weeks | Treatment naïve/PRS-experienced |
| EXPEDITION-1 | GT1, 2, 4-6 | 12 weeks | Compensated cirrhosis  + Treatment naïve/PRS-experienced |
| EXPEDITION-2 | GT1-6 | 8 weeks  12 weeks | HIV-1 co-infection  ± Compensated cirrhosis  + Treatment naïve/PRS-experienced |
| EXPEDITION-4 | GT1-6 | 12 weeks | CKD Stage 4/5 (including on dialysis)^†^  ± Compensated cirrhosis  + Treatment naïve/PRS-experienced |
| GT, genotype; PRS, interferon (IFN)/pegylated (peg) IFN ± ribavirin (RBV or sofosbuvir + RBV ± pegIFN; DAA, direct acting antiviral; HIV-1, human immunodeficiency virus-1; CKD, chronic kidney disease  †Patients with creatinine clearance <30 mL/min were eligible to enroll only in EXPEDITION-4, otherwise all other clinical trials required a screening creatinine clearance ≥50 mL/min | | | |

## Exclusion

1. Female subject who is pregnant, breastfeeding or is considering becoming pregnant during the study; or a male whose partner is pregnant or planning to become pregnant during the study.
2. Recent (within 6 months prior to study drug administration) history of drug or alcohol abuse that could preclude adherence to the protocol in the opinion of the investigator.
3. Subjects on peritoneal dialysis.
4. Positive test result at Screening for hepatitis B surface antigen (HBsAg; for all HCV genotypes 1-6) or Human Immunodeficiency virus (HIV) Ab (for HCV genotypes 2-6).
5. HCV genotype performed during Screening indicating co-infection with more than one HCV genotype.
6. Requirement for and inability to safely discontinue the medications or supplements listed below at least 2 weeks or 10 half-lives (whichever is longer) prior to the first dose of any study drug.
   - Any herbal supplement (including milk thistle), read yeast rice (monacolin K), St. John’s Wort
   - Carbamazepine, phenytoin, pentobarbital, phenobarbital, primidone, rifabutin, rifampin
   - Atorvastatin, lovastatin, simvastatin
   - Astemizole, cisapride, terfenadine
7. Clinically significant abnormalities or co-morbidities based upon the results of a medical history, physical examination, vital signs, laboratory profile, and a 12-lead electrocardiogram (ECG) that make the subject an unsuitable candidate for this study in the opinion of the investigator, including, but not limited to::

- Uncontrolled diabetes as defined by a glycated hemoglobin (hemoglobin A1C) level > 8.5% during Screening.
- Active or suspected malignancy or history of malignancy (other than basal cell skin cancer or cervical carcinoma in situ) in the past 5 years.
- Uncontrolled cardiac, respiratory, gastrointestinal, hematologic, neurologic, psychiatric, or other medical disease or disorder, which is unrelated to the existing HCV infection.

1. Any cause of liver disease other than chronic HCV-infection, including but not limited to the following:
   - Hemochromatosis.
   - Alpha-1 antitrypsin deficiency.
   - Wilson's disease.
   - Autoimmune hepatitis.
   - Alcoholic liver disease.
   - Steatohepatitis on liver biopsy considered to be the primary cause of the liver disease rather than concomitant/incidental with HCV infection.
2. Screening laboratory analyses showing any of the following abnormal laboratory results:
   - ALT > 10 × ULN
   - AST > 10 × ULN
   - Calculated creatinine clearance (using Cockcroft-Gault method) of < 50 mL/min except in EXPEDITION-4
   - Direct bilirubin > ULN
   - Albumin < 3.0 g/dL
   - International normalized ratio (INR) > 1.5 × ULN, unless subject has known hemophilia or is on a stable anticoagulant regimen affecting INR
   - Hemoglobin < 10 g/dL for women; < 11 g/dL for men
   - Platelets < 60,000 cells per mm^3^ for subjects with cirrhosis; < 90,000 cells per mm^3^ for subjects without cirrhosis
3. History of solid organ transplantation.
4. Receipt of any investigational product within a time period equal to 10 half-lives of the product, if known, or a minimum of 6 weeks (whichever is longer) prior to study drug administration.
5. Any current or past clinical evidence of decompensated liver disease such as ascites noted on physical exam, use of beta-blockers for portal hypertension, hepatic encephalopathy or esophageal variceal bleeding.
6. Consideration by the investigator, for any reason, that the subject is an unsuitable candidate to receive ABT-493/ABT-530.
7. Requirement for chronic use of systemic immunosuppressants including, but not limited to, corticosteroids (prednisone equivalent of > 10 mg/day for > 2 weeks), azathioprine, or monoclonal antibodies (e.g., infliximab).
8. History of severe, life-threatening or other significant sensitivity to any excipients of the study drug.
9. Treatment for an AIDS-associated opportunistic infection (OI) (Appendix E) within 6 months of Screening (only in SURVEYOR-I).
10. Patients who cannot participate in the study per local law.

# Supporting Table 2: Treatment Compliance by Number of Psychiatric Co-medications and Treatment Duration

| Characteristic, % (n/N) | G/P Treatment Compliance |
| --- | --- |
| Number of Psychiatric Medications |  |
| 0 | 96.5 (1995/2067)^†^ |
| 1 | 95.4 (334/350) |
| >1 | 95.2 (100/105) |
| Treatment duration |  |
| 8 weeks | 96.0 (926/965) |
| 12 weeks | 96.6 (1388/1437) |
| 16 weeks | 95.8 (115/120) |
| †Includes all patients without a psychiatric disorder and 334 patients who were not taking a psychiatric medication, but were classified as having a history of psychiatric disorder based on 1 or more medical diagnoses | |

# Supporting Figure 1: Subgroup Efficacy Analysis by Baseline Characteristics in Patients with or without Psychiatric Disorder

# Supporting Figure 2: Efficacy by ITT and modified ITT analysis for Patients with or without Psychiatric Disorders by Adherence

# **Supporting Figure 3: Efficacy of G/P in Patients taking a Neuropsychiatric Co-Medication with a Potential DDI by ITT Analysis**

# Supporting Figure 4: Efficacy by ITT Analysis in Patients by Number of Psychiatric Co-medications Taken in Patients with Psychiatric Disorders

**Supporting Table 3: Adverse Events in Patients taking a Neuropsychiatric Co-Medication with a Potential DDI**

| **Event, n (%)** | **Any patients taking Quetiapine**  **N = 47** | **Any patients taking Oxycodone**  **N = 81** | **Any patients taking Hydrocodone**  **N = 77** |
| --- | --- | --- | --- |
| Any AE | 35 (74) | 53 (65) | 58 (75) |
| Serious AE | 1 (2)^†^ | 9 (11) ^‡^ | 5 (6)^§^ |
| DAA-related serious AE | 0 | 0 | 0 |
| AEs leading to discontinuation | 0 | 1 (1)^¶^ | 0 |
| DAA-related AEs leading to discontinuation | 0 | 1 (1) ^¶^ | 0 |
| AE, adverse event; DAA, direct-acting antiviral  † Patient experienced a serious AE of schizophrenia on Day 71 leading to prescription of quetiapine on Day 77 during 12-week G/P treatment  ‡Nine patients experienced 14 serious AEs including arterial injury, suicide attempt, pneumonia aspiration, death (acute ethanol and combined methadone toxicity), left preseptal facial cellulitis, cholecystitis, back pain, infected fistula, catheter site infection, superior vena cava occlusion, gastrointestinal hemorrhage (twice in one patient), pneumonia, ankle fracture, and joint dislocation  §Five patients each experienced 1 serious AE (left preseptal facial cellulitis, worsening of umbilical hernia, parapneumonic effusion, back pain, and femur fracture)  ¶DAA-related non-serious case of diarrhea on Day 27 that led to G/P premature discontinuation that day and subsequent resolution of the AE by Day 32 (6 days after G/P discontinuation) | | | |
